# Epigenetic regulation of macrophages: from homeostasis maintenance to host defense

**DOI:** 10.1038/s41423-019-0315-0

**Published:** 2019-10-29

**Authors:** Siyuan Chen, Jing Yang, Yuquan Wei, Xiawei Wei

**Affiliations:** Laboratory of Aging Research and Cancer Drug Target, State Key Laboratory of Biotherapy, National Clinical Research Center for Geriatrics, West China Hospital, Sichuan University, No. 17, Block 3, Southern Renmin Road, 610041 Chengdu, Sichuan PR China

**Keywords:** Macrophage, Epigenetics, Polarization, Immune memory, Monocytes and macrophages, Infection

## Abstract

Macrophages are crucial members of the innate immune response and important regulators. The differentiation and activation of macrophages require the timely regulation of gene expression, which depends on the interaction of a variety of factors, including transcription factors and epigenetic modifications. Epigenetic changes also give macrophages the ability to switch rapidly between cellular programs, indicating the ability of epigenetic mechanisms to affect phenotype plasticity. In this review, we focus on key epigenetic events associated with macrophage fate, highlighting events related to the maintenance of tissue homeostasis, responses to different stimuli and the formation of innate immune memory. Further understanding of the epigenetic regulation of macrophages will be helpful for maintaining tissue integrity, preventing chronic inflammatory diseases and developing therapies to enhance host defense.

## Introduction

Macrophages are essential phagocytes of the innate immune system and are present in all organs and tissues of the body as resident cells or the differentiation products of recruited blood monocytes.^1^ Macrophages play a critical role in both the maintenance of tissue homeostasis and the regulation of inflammation, completing necessary tissue-specific functions as well as protecting the organism from infection.^2,3^ In addition, macrophages are major producers of cytokines, which are implicated in the pathogenesis of inflammatory disease, and are strategically placed to orchestrate both innate immune responses and adaptive immune responses.^4,5^

Macrophages display high heterogeneity, not only in their inherent terminal differentiation pathway but also in their different responses to different environmental stimuli. Both tissue-resident macrophages in homeostasis and activated macrophages under stimuli are driven by specific transcriptional changes and are controlled by complex cellular mechanisms. Among them, epigenetics now arises as a key controller of macrophage activity.

## Epigenetic modifications

Epigenetics regulates the expression of DNA-encoded information and determines the specific “identity” of a cell while the genetic code is not altered. The chromatin conformation controls transcription factor binding and gene expression through DNA accessibility. Epigenetic markers have traditionally been considered stable and heritable.^6^ However, it has been revealed that epigenetic chromatin markers are dynamically regulated with changes in development or environmental signals.^7^ These results have led to additional information regarding epigenetics, including a transient change in chromatin in response to external stimuli that control gene expression.

Currently, most of the epigenetic mechanisms are related to the protein−DNA interactions that affect gene expression, whether through the recruitment of protein complexes involved in nucleosome modification and remodeling or by chemical modification of DNA bases, such as 5-methylcytosine. Additionally, there are other epigenetic mechanisms, such as the production of small noncoding RNAs, that can regulate mRNA in transcription through base pairing, resulting in mRNA degradation or translational inhibition. Overall, epigenetic changes can be divided into three categories: (1) posttranscriptional histone modifications, (2) DNA methylation, and (3) noncoding RNA.

### Posttranscriptional histone modifications associated with chromatin structure and function

In eukaryotes, approximately 147 bases of DNA wrap around a histone octamer to form a nucleosome.^8^ The octamer, composed of two groups of H2A, H2B, H3 and H4 proteins, is the fundamental unit of chromatin, and histone H1 connects the two nucleosomes. When nucleosomes are organized into tightly arranged bundles (heterochromatin), transcription is inhibited by restricting the entry of transcriptional machinery. In contrast, when the chromatin is relaxed (euchromatin), the nucleosomes are arranged like a string of beads, predominantly associated with active transcription. Nucleosome-free regions are known as open chromatin, which contain a variety of functional sequences, such as promoters and enhancers, and thus play a vital role in gene regulation.^9^ Promoters are short sequences of approximately 100 bases proximal to the transcription start sites at the 5′ ends of genes. A common feature of promoters is that RNA polymerase II binds to promoters of inactive genes in advance. When stimulated, promoters can recruit more polymerase II to initiate transcription.^10^ Enhancers are distal transcriptional regulatory elements that are defined as genomic DNA elements, ranging from several to hundreds and, in rare cases, even thousands of bases in length, and contain short transcription factor (TF) recognition sequences or binding sites. In these regulatory regions, the accessibility of DNA by protein TFs and other transcriptional mechanisms is critical for gene expression. The interplay and long-range communication of enhancers and target gene promoters mediated by DNA looping tightly regulate gene expression.

Disordered N-terminal histone tails extending from nucleosomes are substrates for a variety of posttranscriptional enzymatic modifications. Amid a vast repertoire of histone modifications, acetylation/deacetylation and methylation/demethylation of lysine residues belong to the most broadly studied and extensively characterized macrophage-related epigenetic modifications of posttranslational histone modification. Determined by the opposing activities of enzymatic writers and erasers, the presence of histone modifications on the chromatin is reversible.

Histone acetyltransferases promote acetylation, which is related to transcriptional activity, such as H3K9ac and H3K27ac, whereas histone deacetylases (HDACs), the erasers, catalyze the removal of acetyl groups from histone tails, which is linked to transcriptional inhibition.^11^ Histone acetylation markers are specifically recognized and bound by bromodomains, a structural motif present in specialized chromatin reader proteins that reads the histone acetylation code, and then transcriptional mechanisms are initiated.^12^ Many histone acetyltransferase complexes contain a component with a bromodomain that anchors histone acetyltransferase complexes on the already acetylated chromatin, enabling them to propagate acetyl labels to adjacent nucleosomes, hence spreading histone acetylation. Similarly, the SWI/SNF complex, the first described ATP-dependent chromatin remodeling complex, targets the acetylated histone tails through the bromodomain subunit, which is critical for recruitment.^13^ Another group of histone acetyl readers, known as the bromo- and extraterminal (BET) protein family members, can recruit TFs and chromatin remodeling complexes to regulate gene expression, and they have been demonstrated to play a key role in the regulation of inflammatory gene expression in macrophages.^14^

Similar to histone acetylation, the subsequent methylation and demethylation of histones are promoted by histone methyltransferases and histone demethylases, respectively. Specifically, every site-specific mark of lysine within the tail of H3 is written by a group of specific enzymes called histone lysine methyltransferases (KMTs) and erased by other enzymes classified as histone lysine demethylases. Unlike acetylation, methylation modification of histone can induce transcriptional activation or inhibition, depending on the location and number of methyl groups within the histone tail. Regulatory elements on repressed genes, such as methylation at the H3K9 and H3K27 loci, often lead to “gene silence” and induce the heterochromatin state in chromatin regions. Conversely, methylation at H3K4 and H3K36 is associated with the assembly of transcriptionally permissive chromatin structures and active transcription at many loci.^15^ Furthermore, the extent of individual lysine methylation, including the mono- (m1), di- (m2), or trimethylation (m3) of histone lysine residues, is differentially distributed in chromatin and plays different roles in gene regulation. Transcriptional activity is correlated positively with the trimethylation of H3 histone lysine 4 (H3K4m3) at gene promoters.^16^ On the other hand, the monomethylation of this residue (H3K4m1) is a typical feature of enhancers.^17^

### DNA methylation in the regulation of gene expression

In addition to histones, methyl modification of DNA is a unique mechanism in epigenetic regulation. In mammals, most DNA methylation occurs in the 5′-cytosine-phosphate-guanine-3′ dinucleotide (CpG) dinucleotide clusters named CpG islands, which are found in the promoters of approximately 40% of the genes, and greater than 70% of CpG sites are methylated in the DNA of somatic cells.^18^ DNA methylation is mainly related to transcriptional inhibition, characterized by 5-methylcytosine immediately adjacent to guanine residues, and involves the transfer of a methyl group to the cytosine ring of DNA through DNA methyltransferases (DNMTs).^19^ Mechanically, 5-methylcytosine binding proteins induce the recruitment of repressor complexes to methylated promoter segments and cause transcriptional silencing.^18^ In particular, DNMT3A and DNMT3B are responsible for establishing de novo methylation markers, while DNMT1 is the maintenance methyltransferase responsible for maintaining these markers, as these markers at CpG dinucleotides must be reestablished after DNA replication in each cell division.^20^ DNMT1 can also participate in the regulation of histone modifications that result in a depletion of di- (H3K9me2) and trimethylation (H3K9me3) at H3K9 and a concomitant increase in H3K9 acetylation (H3K9ac).^21^

Recently, the methylation of non-CpG moieties has been found to occur widely on genomic DNA.^22^ The abundant non-CpG methylations of DNA directly affect the binding of TFs through the methylation of TF binding sites, which provide a mechanism for regulating gene expression.^22^

### Noncoding RNA in posttranscriptional regulation

Finally, endogenously expressed noncoding RNAs, including long noncoding RNAs (lncRNAs) and microRNAs (miRNAs), play critical roles in the posttranscriptional regulation of gene expression by acting as competing RNAs, although they do not directly affect chromatin structure. lncRNAs are a large group of nonprotein-coding transcripts with a length of more than 200 nucleotides. XIST, one of the best characterized lncRNAs, was found to drive X-chromosome inactivation in 1991.^23^ Thereafter, thousands of lncRNAs have been found in different cells and to be involved in a variety of diseases. Although the sources of these lncRNAs are different, their functional mechanisms are similar. A number of lncRNAs can directly interact with chromatin-modifying enzymes and remodeling complexes to guide these epigenetic catalysts to specific parts of the chromatin.^24^ In addition, some lncRNAs can form RNA−protein complexes with transcription factors and affect the location and activity of transcription factors binding to them, thereby regulating gene expression.^25^ Specific lncRNAs play a part in the programming of silencing or activating histone modifications. For example, HOTAIR, a 2.2 kilobase lncRNA in the HOXC locus, is involved in silencing chromatin mediated by polycomb repressive complex 2 (PRC2, comprised of SUZ12, EED, and the lysine methyltransferase EZH2) and can recruit PRC2 to its target gene and methylate H3K27 via EZH2.^26^ Furthermore, HOTAIR has been found to be a scaffold that targets genes in at least two different histone modification complexes.^27^ The 5′ domain of HOTAIR binds to PRC2, and the 3′ domain of HOTAIR binds to the CoREST/REST repressor complexes, which contain LSD1 (KDM1/BHC110), a demethylase that mediates the enzymatic demethylation of H3K4me2. The ability to link two distinct complexes makes it possible to assemble PRC2 and LSD1 and coordinate the complexes targeting chromatin to achieve the methylation of H3K27 and the demethylation of H3K4.^27^ In addition, another typical example is the lncRNA ANRIL, an antisense RNA transcript overlapping the INK4b/ARF/INK4a tumor suppressor locus, which participates in the *cis* recruitment of both PRC1 and PRC2 to the target gene for silencing.^28^

Another class of noncoding RNAs, miRNAs, which are single-stranded RNAs containing 20−24 bases. It is estimated that more than 60% of all protein-coding genes are directly regulated by miRNAs. They can specifically bind to the 3′ or 5′ untranslated regions of mRNA, mainly as posttranscriptional inhibitors, by targeting the 3′ untranslated regions of the RNA to stimulate its degradation and translation repression.^29^ In addition, a specific miRNA may bind to and regulate several targets, sometimes as part of the same signaling pathway, adding multiple regulatory levels. Therefore, under pathophysiological stimulation, miRNAs can fine-tune gene expression patterns.

### Crosstalk mechanisms

Based on existing research, mechanisms of crosstalk among the different epigenetic regulation systems have been revealed. They interact with each other rather than working independently. For example, histone lysine is the target of both methylation and acetylation, such as H3K9 and H3K27. At least two enzymatic steps are required to shift from the inhibited methylated state to the activated acetylated state (and vice versa). Therefore, it is believed that the state between the opposite nucleosome-modifying activities is altered by sequence-specific DNA-binding TFs that recruit specific chromatin remodeling agents and modifying factors to individual loci, and crosstalk between them occurs, leading to their combinatorial effects in transcriptional control.^30^ Histone and DNA modification, together with lncRNAs and miRNAs, are collectively defined as epigenetic mechanisms.

## Epigenetic mechanisms of macrophages in tissue homeostasis

### Origin of macrophages

In 1968, it was presumed that macrophages were derived only from blood monocytes, and this concept prevailed for approximately half a century.^31^ Accumulating evidence, however, has demonstrated that the different macrophages do not necessarily have the same origins. With few notable exceptions, the tissue macrophages do not arise from blood monocytes under homeostasis and even in some kinds of inflammation; those tissue macrophages are seeded from embryonic precursors of the yolk sac macrophages and fetal monocytes prior to birth and maintain themselves throughout adulthood by self-renewal and participate in tissue remodeling. Erythro-myeloid progenitors, derived from the yolk sac, have been found to be the embryonic precursors of yolk sac macrophages and fetal monocytes before the emergence of hematopoietic stem cells (HSCs); thus, they are a common origin for tissue macrophages.^32^ Two sequential waves of erythro-myeloid progenitors were identified to generate macrophages. An early myeloid-restricted hematogenic wave originates in the yolk sac, generates primitive macrophages without monocytic intermediates and is independent of the transcription factor c-Myb.^33^ These cells either directly generate primitive yolk sac macrophages as the sole origin of tissue-resident macrophages, such as microglia, or migrate to the fetal liver and give rise to fetal monocytes that proliferate in the embryonic tissues and differentiate into distinct tissue macrophages.^34^ Alternatively, a late c-Myb-dependent wave that commences in the fetal liver to generate multiple hematopoietic lineages, including monocytic intermediates, persists throughout adult life.^35^ Fetal monocytes differentiate into macrophages when recruited to fetal tissues, replacing the main components of the existing yolk-sac-derived macrophages gradually with the exception of microglia.^35^ With the growth of host tissues, these primitive macrophages differentiate through proliferating in their respective tissue macrophage compartments in most tissues, except the intestine, the heart, and the skin. Therefore, a model of macrophage origination has been established, which is distinct from the model that depends only on blood monocytes.

### Role of the lineage-determining factor PU.1 and enhancers in macrophages

Tissue-resident macrophages are an extremely heterogeneous population, which is a necessary outcome of lineage- and tissue-specific functions during development and adulthood, and are integral to maintenance of tissue homeostasis. Independent of each other, tissue macrophage compartments evolve locally surrounded by their organ microenvironment, and therefore, each population of tissue macrophages is closely related to its immediate surroundings. Hence, these cells acquire additional functions and activities tailored to maintain the steady state in local tissues through various functions, including the modification of the phagocytosis mechanism, general or specific niche nutritional factors, and morphological specificity.

Normal tissue homeostasis is regulated in a tissue-specific manner by distinct populations of tissue macrophages, ranging from pulmonary surfactant clearance to neuron pruning and the establishment of intestinal homeostasis. Epigenetic characterization shows great plasticity in the epigenetic programs of macrophages, and more than 12,000 enhancers can be reprogrammed when mouse differentiated macrophages are transplanted into a new microenvironment.^36^

Enhancers are fundamental and precise determinants of gene expression and play a key role in how distinct signals establish cell identity and regulatory potentiality at the genomic level. The number of enhancers identified in murine macrophages exceed that of promoters; thus, interactions between DNA and TFs at enhancers are more likely to occur than at promoters.^37^ Importantly, there is strong enrichment for sequences containing transcription factor binding sites that recognize and recruit different patterns of TFs on macrophage enhancers, corresponding to a significant enrichment of DNA recognition motif combinations.^38^ In turn, the binding of TFs to DNA determines the selection of new enhancers.

**Fig. 1:**
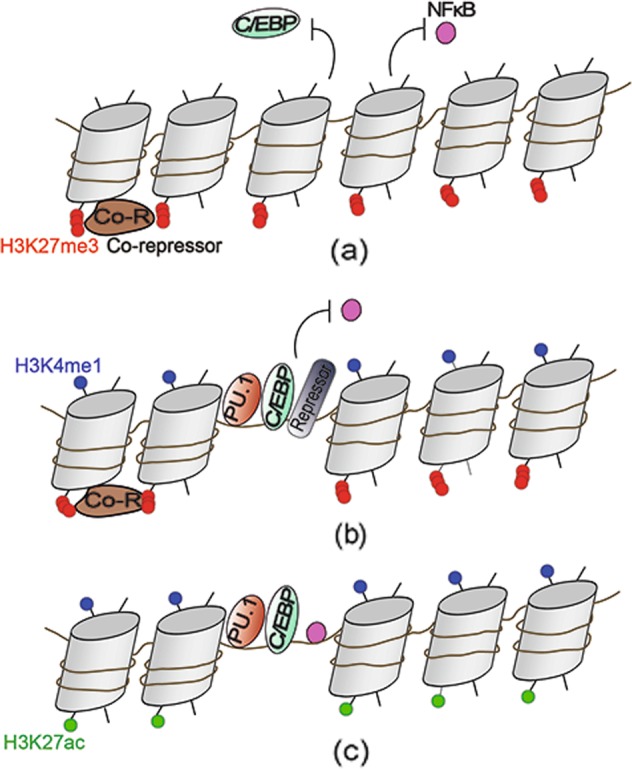
Epigenetic lineage determination and signal stimulation collaboratively control the enhancers of macrophages. **a** Without the expression of the master macrophage regulator PU.1, cells do not receive genomic signals not relevant to their own functions. The gene loci exhibit inaccessible chromatin, the suppressive histone marker H3K27me3 and the occupancy of corepressors. **b** During lineage establishment, the nucleosome in macrophages is evicted more effectively as a result of a relatively prevalent binding of PU.1 that unpacks the tight organization of chromatin. Collaborative TFs, including C/EBP, are subsequently recruited to establish macrophage enhancers. Enhancers are commonly marked by the epigenetic signature H3K4me1. Without activating factors, the genes are poised at baseline, meaning that the enhancers are also marked by H3K27me3 and the binding of repressor complexes. **c** The presence of local signals, such as TLR4 stimulation, for example, allows for the efficient binding of NF-κB. In addition, the genes lose the H3K27me3 mark on the enhancers and are acetylated at H3K27. These mechanisms facilitate related gene transcription

In addition to tissue-specific regulation, which is very predictable, to be more precise, the epigenetic regulation of macrophages is coordinated by lineage- and tissue-specific transcription factors, which are determined by the built-in programming of myeloid development as well as signals from the tissue environment. Lineage-determining transcription factors (LDTFs), also referred to as pioneer factors or master regulators, can actively open up the local chromatin and directly combine it with other factors.^39^ Multiple lines of evidence suggest that ETS family member PU.1 contributes to the basal activation state and H3K4me3 of many promoters, acts as a crucial LDTF that occupies most macrophage enhancers where it is essential in maintaining H3K4me1, and contributes to select a large number of the cell-specific enhancer-like elements, suggesting that PU.1 is both required and sufficient to function in genomic regions as an enhancer.^40^ Moreover, PU.1 is considered to be a pioneer TF in initiating chromatin accessibility, allowing the binding of additional TFs, and PU.1 is bound at the same level in both unstimulated and stimulated cells. Additional TFs commonly found in macrophages, including C/EBP family members, IRF, NF-κB and AP-1 factors, exhibit collaborative interactions with macrophage-specific enhancers selected by PU.1 (Fig. 1).^41^ Studies have validated that the collaborative and hierarchical relationship of LDTFs and signal-dependent transcription factors (SDTFs) exist at pre-existing enhancers.^41^ Mutations in PU.1 motifs leading to the inhibition of PU.1 binding cause the deletion of the binding of C/EBPα in collaboration. Conversely, mutations in C/EBP motifs also result in a corresponding reduction in nearby PU.1 binding. Moreover, mutations in LDTF motifs can abolish the signal-dependent binding of NF-κB, whereas mutations in NF-κB motifs rarely affect the binding of PU.1 or C/EBPα. Therefore, the hierarchical model of regulatory functions in macrophages has been described, where a relatively small group of LDTFs compete with nucleosomes to bind DNA in a cell-specific manner, among which PU.1 is a necessary LDTF of macrophages to maintain nucleosome depletion at macrophage-specific enhancers.

By initiating nucleosome remodeling and histone modification deposition that are associated with *cis*-active regulatory elements, the binding of PU.1 is proposed to “prime” DNA, and thus, the common enhancer repertoire is activated differentially by H3K4 monomethylation at a large number of genomic regions.^40^ Simultaneously, or subsequently, the binding of secondary SDTF establishes tissue-specific enhancers and regulates gene expression.^42^ Cooccurrence motifs associated with these TFs can be traced back to the fact that enhancers provide integration sites of TFs regulated at the genome level by internal and external environment signals. Accordingly, synergistic binding of TFs can promote the ability to overcome a nucleosomal barrier and initiate chromatin regulatory events. In this way, tissue-specific TFs and tissue-programmed epigenetics of distinct tissue environments control the gene expression of resident macrophages, regulating their functions and affecting the environment itself.

While some lineage-specific enhancers are “open” and labeled by H3K4me1 in HSCs, it seems that a considerable number of de novo establishments of enhancers occur during hematopoiesis. For instance, many macrophage-specific enhancers are established in granulocyte-macrophage progenitors (e.g., H3K4me1) but are blocked in HSCs, such as the ones that drive the expression of the gene encoding CD11b, which is the integrin and common myeloid marker.^43^ Particularly, NF-κB also shows the ability to select “latent” or “de novo” enhancers in cooperation with PU.1 to bind to genomic locations lacking prior characteristics associated with active enhancers.^44^ In fact, a promoter can be affected by a variety of different combinations of enhancers with different SDTF motifs that are intrinsically related to various signaling pathways. This feature of enhancers constitutes great flexibility in tuning gene expression according to the specific needs that macrophages encounter in a context-dependent manner.

Generally, active enhancers contain both H3K4me1 marks and H3K27ac marks, while enhancers in the poised state are marked by H3K4me1 and in the absence of the activating acetylation marker H3K27ac.^45^ These poised enhancers shared by many tissue macrophages are not active but might indicate the potential to respond to local challenges and activate prospective gene-expression programs.^46^ The research on promoters (H3K4me3^+^), poised enhancers (H3K4me1^+^ and H3K27ac^−^) and activated enhancers (H3K4me1^+^ and H3K27ac^+^) has suggested that, independently of development, signals from the local microenvironment play a leading role in the formation of tissue-specific regulatory regions and in the control of gene activity in macrophages.^36^

### Significant effects of macrophages in specific tissues

**Fig. 2:**
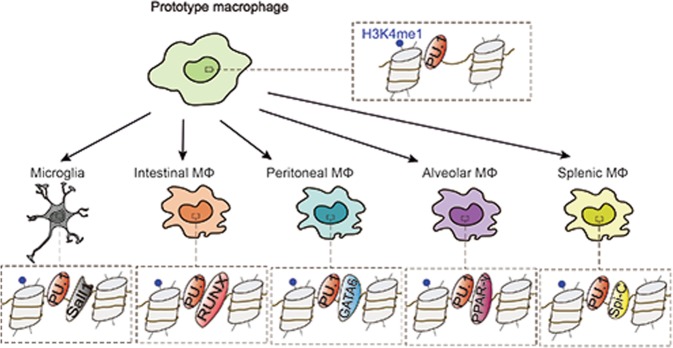
Tissue macrophages are developed alongside distinct environment-specific signal-dependent transcription factors. The model shows the development of different tissue macrophages. A prototype macrophage has a PU.1-bound chromatin landscape, and the naïve landscape is exposed to distinct environmental signals in each tissue. As a result, tissue-specific enhancers regulate unique gene expression

The existence of multiple macrophage progenitors begs the question of whether the ontogenesis of macrophages determines their functional characteristics. In fact, distinct macrophage populations display unique transcriptional characteristics and epigenetic marks that are specific to their tissue of residence (Fig. 2).

Microglia, the macrophages of the central nervous system (CNS), are resident macrophages in the brain and spinal cord that are entirely derived from the yolk sac during embryogenesis, potentially and specifically due to their privacy behind the blood−brain barrier.^34^ Microglia play diverse roles in the healthy brain, from forming developing neuronal circuits to shaping learning-related plasticity.^47^ In healthy brains, microglia are highly active and constantly observe the microenvironment with extremely motile processes and protrusions. The transcriptomic and epigenetic phenotypes of microglia are relatively well conserved.^48^ Snall1 is a microglial unique TF, and its enhancers are open and active only in microglia.^36^ Therefore, Snall1 controls the transcriptional regulation that maintains microglial identity and physiological properties as a critical factor for CNS homeostasis.^49^ Recently, the functions of HDAC1 and HDAC2 in microglia have been found to be time- and context-dependent in vivo.^50^ Prenatal ablation of HDAC1 and HDAC2 results in spontaneous microglial damage due to abundant H3K9ac and H3K27ac deposits on their respective promoters, but the lack of HDAC1 and HDAC2 enhances the phagocytic function of microglial amyloid proteins during neurodegeneration.^50^ In addition, miR-124, a brain-specific microRNA expressed in microglia but not in peripheral macrophages, has been detected to be a crucial regulator for maintaining the quiescent state of microglia in the CNS.^51^ In terms of mechanism, miR-124 can reduce the expression of PU.1 and its downstream target, the M-CSF receptor; suppress cell proliferation; and enhance the differentiation of primary macrophages into adult microglia via the C/EBP-α–PU.1 pathway.^51^ Blood−brain barrier breakdown is associated with brain pathologies and induces prominent monocyte infiltration into the CNS. The monocytes give rise to brain macrophages and can hardly be discerned from resident embryo-derived microglia.^52^ Microglia and monocyte-derived cells have distinct functions, as revealed by the study of murine multiple sclerosis and the experimental autoimmune encephalomyelitis model.^53^ During encephalomyelitis, microglia appear dedicated to the clearance of debris, whereas monocyte-derived macrophages are highly phagocytic and inflammatory, with the expression of proinflammatory genes. Specifically, monocyte-derived macrophages are unable to generate long-lived microglia and do not permanently contribute to the brain-resident macrophage compartment, which is thus composed of embryo-derived microglial cells before and after the challenge.^54^

The intestine contains the largest pool of macrophages among the tissues.^55^ As the extreme opposite of CNS microglia, embryo-derived macrophages are found in the intestine only shortly after birth. While embryonic precursors seed the intestinal mucosa and demonstrate extensive proliferation in the neonatal period, these cells do not appear in the adult intestine.^56^ Instead, they are replaced entirely around the time of weaning by the blood inflammatory monocytes that migrate to inflamed tissues, differentiate locally into mature tissue macrophage populations in healthy intestinal lamina propria and contribute to the maintenance of gut homeostasis. Intestinal macrophages are enriched for RUNX family motifs, and RUNX3 is highly expressed in these cells.^36^ The monocyte infiltration is regulated mainly by the microbiota, which leads to low-grade inflammation. In fact, intestinal macrophages spend their existence bathed in cytokine IL-10 and maintain anti-inflammatory responses that mute any inflammatory response to the gut flora and their products.^57^ This progress can eliminate the pathogen, avoid collateral damage caused by the oversecretion of proinflammatory cytokines and restore tissue integrity as a result.

Alveolar macrophages (AMFs) located in the alveolar cavity develop from fetal liver monocytes depending on CSF2 (also known as GM-CSF) through the induction of peroxisome proliferator-activated receptor-γ (PPAR-γ).^58^ Before birth, embryo-derived primitive macrophages and fetal monocytes are colonized in the developing lung.^59^ The initial signs of AMF differentiation appear around the saccular stage of lung development, and it is assumed that fetal monocytes, but not embryo-derived macrophages, are the main precursors of AMFs. With minimal contribution from circulating hematopoietic precursors, AMFs self-maintain locally. After birth, AMFs play an important role in the scavenging of lung surfactant and pulmonary homeostasis. Transcriptional inhibitors Bach1 and Bach2 are required for functional maturation; Bach2 in particular is a major contributor to this repression.^60^ The lncRNA MEG3-4 has been identified as a tissue-specific regulator of inflammatory responses in alveolar macrophages during bacterial infection through the transcriptional regulation of immune response genes.^61^ It has been confirmed that the lncRNA MEG3-4 binds to the microRNA miR-138 in a competitive manner with mRNA encoding the proinflammatory cytokine IL-1β, thereby increasing the abundance of IL-1β and enhancing the inflammatory response to bacterial infection in alveolar macrophages in mice.^61^

The spleen-macrophage chamber actually contains several different compartments, including the marginal zone (MZ), red pulp (RP), and white pulp (WP) subtypes, which have independent and noncomplementary functions that are linked to the phagocytic capacity of specific macrophage subpopulations.^62^ Macrophages located near the splenic MZ, the channel through which the bloodstream passes, seem to be uniquely committed to the stationary clearance of apoptotic cells and the selective engulfment of dying cells.^63^ In the MZ, the nuclear receptor LXRA is essential for macrophage differentiation, as proven in LXRA-deficient mice, which have defective generation of the MZ and metallophilic macrophages.^64^ In contrast, macrophages in the RP specifically remove aging or damaged red cells and recover the released iron. It has been discovered that the transcription factor Spi-C, a PU.1-related transcription factor, selectively dominates the development of red pulp macrophages.^65^ Due to the lack of Spi-C, a deficiency of macrophages in the red pulp impairs the clearance of senescent red blood cells and the maintenance iron homeostasis, and selective splenic iron overload occurs in mice with this deficiency.^65^

As for peritoneal macrophages, there are at least two distinct macrophage subtypes in the abdominal cavity of adult mice.^66^ In healthy mice, “large” peritoneal macrophages account for the principal part of the components of peritoneal cavity macrophages but disappear rapidly after stimulation. In addition, “small” peritoneal macrophages, as a source of controversy, predominate in the peritoneal cavity after stimulation. The phagocytosis of these macrophages eliminates apoptotic cells. Evidence suggests that retinoic acid is the tissue-derived signal that induces the localization and functional polarization of peritoneal macrophages in a tissue-specific manner through the reversible induction of GATA6, a specific TF for peritoneal macrophages associated with the establishment of the tissue-specific transcriptional and epigenetic landscape.^67^ Moreover, previous research has shown that DNA methyltransferase DNMT3A maintains a high expression of HDAC9 in a DNA-methylation-dependent manner in naïve peritoneal macrophages and epigenetically prepares these cells to activate TBK1-IRF3 signaling fully and produce interferon I after virus infection.^68^

As mentioned above, macrophages promote tissue homeostasis under physiological conditions in distinct manners. In a stable state, tissue macrophages have intrinsic and potential anti-inflammatory functions. Tissue-resident macrophages derived from the yolk sac, fetal monocytes and adult monocytes all exhibit inhibitory effects despite differences in ontogeny. Immunosuppression may be the key function of macrophages in tissue homeostasis. Nevertheless, macrophages in homeostasis are primed to respond rapidly and robustly to subsequent challenges, maintaining low levels of constitutive IFN-β and downstream Janus kinase (JAK)–STAT signaling.^69^ Two almost simultaneous studies introduced the importance of commensal microbiota in the production of low levels of IFN.^70,71^ It is particularly noteworthy that the microbiota mimics the regulatory components of host protein networks. For example, influenza A virus carries a sequence that resembles H3K4 and can block interactions with readers of H3K4me3, thereby suppressing the positive function of this epigenetic marker.^72^ Another mechanism that primes macrophages is the maintenance of low levels of negative H3K9me3 marks at IFN response gene loci.^15^

### Epigenetic variations in different tissue-resident macrophages

As mentioned above, analyses of enhancer landscapes have revealed that some enhancer-like regions in various tissue-resident macrophage populations are shared and that the combination of PU.1 is required for the development of macrophages in almost all tissues. The enhancer of Spi1, which controls the expression of PU.1, has H3K4me2 and H3K27ac marks in all macrophage populations.^18^ In contrast, there are epigenetic variations in macrophage populations in different tissues. For example, the Rarb gene, induced by retinoic acid, is labeled by H3K4me2 in all macrophage populations, but H3K27ac only presents in the peritoneal macrophage population.^42^ The fact that the enhancer region of the Rarb gene is poised in other tissue-resident macrophages but active in peritoneal macrophages is consistent with another finding that peritoneal macrophages are controlled by locally produced retinoic acid, and the Rarb gene itself is retinoic acid inducible.^73^ This evidence suggests that the enhancers leading to Rarb expression are selected by a series of common macrophage lineage-determining factors in other macrophages and peritoneal macrophages. However, these enhancers can become active only in the peritoneal cavity because of the sufficient concentrations of retinoic acid in the tissue environment.

## Epigenetic regulation of macrophage activation in host defense

### Different polarization states of active macrophages

In addition to maintaining tissue homeostasis induced by homeostatic signals, macrophages are best known for their role as immune guards at the forefront of tissue defense. In response to various external stimuli and diverse signals, macrophages can obtain heterogeneous activation states and customize specific functions for specific microenvironments. The accurate and specific regulation of macrophage activation to eliminate the pathogenic insult and repair damaged tissue, in turn, is crucial for restoring tissue homeostasis. As shown previously, tissue-specific phenotypes of macrophages, controlled by lineage-specific master regulators, can be generated by hard-wired, irreversible differentiation processes. Alternatively, phenotypes can be reversible and induced as needed based on a functional polarization program.

From the previous literature, two extreme states of activated macrophages are M1-like macrophages (in response to inflammatory stimuli such as LPS and IFN-γ) and M2-like macrophages (in response to cytokines such as IL-4 and IL-13).^74^ In addition, the growth factors GM-CSF and M-CSF, traditionally used to differentiate monocytes into dendritic cells or macrophages, have the ability alone to induce M1- and M2-like phenotypic changes, respectively.^75^ In fact, the phenotypes of macrophages can switch between different functional states, and the stability of M1, M2 and other phenotypes is unclear. M1 and M2 are more accurate when describing macrophages in vitro, which are induced by specific stimuli. However, in vivo macrophages have heterogeneous phenotypes and multiple polarization states, and thus, they can hardly be simply binned into an M1 or M2 pool. The description of the activation states of these macrophages is currently contentious and confusing. Nonetheless, it is still useful to conceptualize these states as they are still related to the binary M1/M2 classification, and the consensus on the definition offers a reductionist tool to describe extremes of their function and may facilitate the study of macrophages.

M1 macrophages, also known as classically activated macrophages, are inflammatory macrophages characterized by efficient antigen presentation, high bactericidal activity, and the production of proinflammatory cytokines and reactive oxygen and nitrogen species.^76^ In contrast, M2 macrophages, often referred to as alternatively activated macrophages, have immune-regulatory functions by releasing anti-inflammatory cytokines and decreasing the production of proinflammatory cytokines and show less efficient antigen presentation. In addition, M2 macrophages are predominantly regulatory macrophages involved in tissue remodeling, tumor progression, wound healing, angiogenesis, anti-helminth responses and allergic reactions.

When early warning signals are triggered, a defined feature of M1 activation is monocyte recruitment from the blood.^77^ Nevertheless, M2 macrophages accumulate independently of monocytes, and in situ proliferation occurs rapidly to induce the accumulation of macrophages under the control of IL-4.^78^

### Monocyte−macrophage transition under stimulation

Monocyte-derived macrophages exist in some tissues, such as the dermis and intestine, where monocytes are considered an intermediate developmental stage between bone marrow precursors and tissue macrophages.^79^ In addition, blood monocytes migrate to inflammatory tissues and differentiate into monocyte-derived macrophages that can restore tissue integrity and eliminate the pathogen.

In vitro, monocyte cultures imitate inflammatory macrophages in vivo and are used clinically as a crucial tool in basic research. Epigenetically, active DNA demethylation is involved in the entire process of monocyte differentiation into macrophages as a major example of the role of targeted demethylation in cell differentiation.^80^ DNA demethylation affects a small group of specific genes that regulate the actin cytoskeleton and phagocytosis and is therefore very important for the structure and function of macrophages.^80^ Notably, these regions become nucleosome-free and obtain active enhanced markers through enrichment in the binding sites of transcription factors (such as AP-1, RFX1 and KLF4), which can open chromatin, and demethylation catalyzed by 10−11 translocation proteins occurs rapidly.^81^ A recent study revealed that during macrophage development, the ATP-dependent chromatin remodeling SWI/SNF complex (also termed BAF), utilizing two alternative ATP-dependent enzymes, brahma-related gene 1 (BRG1) and brahma (BRM), activates the expression of DNA repair enzymes by recognizing and replacing epigenetically marked nucleosomes, together with EP300 and HDAC1, constituting a functional unit.^82^ Along the monocyte−macrophage differentiation axis, EP300-HDAC-SWI/SNF functional crosstalk defines the chromatin structure and transcriptional activity of DNA repair enzyme promoters.^82^

Recently, high-throughput epigenome analysis has revealed that three miRNAs (miR-34, miR-146 and miR-221), which play essential roles in actin cytoskeletal reorganization, are upregulated and overexpressed in the process of monocyte-to-macrophage differentiation.^80^ In contrast, other miRNAs have been found to be downregulated during monocyte-to-macrophage differentiation. miR-198, which is capable of reducing cyclin T1 protein expression through translational inhibition, for instance, is strongly downregulated during this process.^83^ The decrease in miR-198 level during macrophage differentiation is beneficial for cyclin T1 protein expression, which has been shown to be important in the regulation of macrophage gene expression.^84^

### Initial inflammatory activation by TLRs

**Fig. 3:**
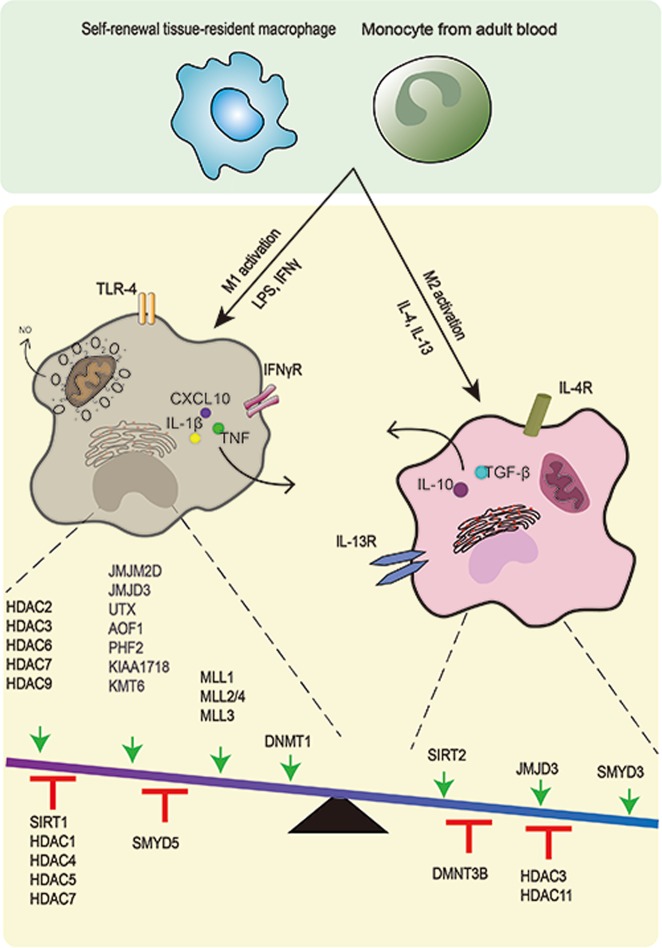
In response to diverse signals, macrophages attain heterogeneous activation states and develop customized, specific functions. Macrophages are activated or polarized by various stimuli, which result in different phenotypes. LPS/IFN-γ leads to inflammatory macrophages, while IL-4/IL-13 induces macrophages to an anti-inflammatory state. The relevant epigenetic enzymes that regulate the macrophage phenotype are summarized by their influence. As shown in the balance model, the enzymes above the arrows have been shown to have activating effects, while those under the T-shaped support stand have repressive effects on M1/M2 activation

Toll-like receptor ligands, such as LPS, and Th1 cytokines, including IFN-γ, elicit M1 activation alone or in combination and can affect epigenetic processes and lead to epigenetic modifications (Fig. 3).^85^ Toll-like receptors (TLRs), especially TLR4, are key sensors in the M1 response that triggers cascades of signals to activate inflammatory processes through MAPK, NF-κB and IRF gene networks, which have downstream genes that encode inflammatory cytokines, such as CXCL10, IL-1β, IL-6, IL-12 p40 and TNF.

LPS affects diverse sets of epigenetic factors that determine the modifications of chromatin structure and gene-expression programs, such as DNA methylation, chromatin-associated complexes, and histone modifications. Until now, however, histone modifications in response to LPS have been studied more extensively. The first evidence on the link between LPS stimulation and epigenetic regulation in inflammatory genes dates back to 1999, as LPS stimulation induces IL-12 p40 production in murine primary activated macrophages by rapid and specific nucleosome translocation at the promoter region.^86^ A subsequent study revealed that it is a TLR-4-dependent event and depends on histone H3 and H4 acetylation.^87^

Prior to the activation of differentiated macrophages, the locus that encodes inflammatory genes appears to be in a relatively “open” chromatin environment, and the epigenetic landscapes are established during macrophage differentiation as stated earlier. As mentioned above, macrophage master TFs, such as PU.1 and C/EBP family members, bind to and open the enhancers of these genes and thus “prime” them. Enhancers in this state are marked by PU.1, H3K4me1, and open chromatin.^40^ In addition, there are gene-specific repressive mechanisms that restrain inflammatory cytokine gene transcription in the absence of TLR signaling. These two opposite effects keep macrophages in a “poised” state. One of the repressive mechanisms is that negative histone marks H3K9me3, H3K27me3, and H4K20me3 are present in the inflammatory gene loci.^88–90^ Another mechanism is the occupancy of gene loci by repressors such as nuclear receptors that recruit corepressor complexes that contain sequence-specific transcriptional repressor B cell leukemia 6 (Bcl6).^91^ In addition, the occlusive positioning of nucleosomes limits the chromatin accessibility of genes such as IL-12b.^10^

Furthermore, these epigenetic “brakes” associated with transcriptional silencing are released after the stimulation of macrophages by TLR ligands. The corepressors are removed from gene loci, and the concomitant reduction of negative histone marks trimethylations by using demethylases. JMJD3, which specifically demethylates trimethylated lysine 27 (H3K27me3), could be induced by NF-κB rapidly under LPS stimulation and recruited to the promoter regions of more than 70% of LPS-induced genes in macrophages and regulate gene transcriptional elongation associated with the H3K27 demethylase KIAA1718.^92,93^ The induction of inflammatory cytokines by acute-phase protein serum amyloid A depends on JMJD3 activity.^94^ Only a subset of proinflammatory cytokines such as IL-12b and CCL5, however, shows a strong dependence on JMJD3 demethylase activity to achieve full activation.^92^

After removal of negative histone marks of inflammatory genes, positive histone marks, such as H3K4me3 and H3K27Ac, increase. The histone marks H3K4me3 are enriched in the promoter region of the M1 marker gene CXCL10 by histone methyltransferase myeloid lymphoid leukemia (MLL), which is shown to increase upon LPS- and IFN-γ-induced M1 activation of macrophages.^95^ Moreover, different kinds of MLLs, such as MLL1, MLL2/4, and MLL3, are critical for the remodeling of the enhancer landscape of macrophage activation.^44^ In addition, a new study suggests that the methyltransferase Dot1l increases the production of IL-6 and TNF-α by promoting H3K79me2/3 and H3K79me2 at their promoters, respectively.^96^

HDACs are strongly involved in M1 activation and play a prominent role in the regulation of immunological pathways. The expression of almost all classes of HDACs is affected upon LPS stimulation, and the sequential regulation of HDAC controls gene-specific expression (Fig. 3). As a typical example, cyclooxygenase-2, a crucial enzyme involved in the inflammatory reaction, is rapidly induced in macrophages upon LPS stimulation, and a number of HDACs are involved in the process.^97^ It has been shown that LPS inhibits the expression of HDAC4, 5 and 7 at first and then upregulates the acetyltransferase complex, leading to cyclooxygenase-2 gene activation.^98^ During *L. pneumophila* infection of pulmonary epithelial cells, a similar mechanism has been described for IL-8 induction, accompanied by the initial decrease in HDAC1 and HDAC5.^99^ Other studies have shown that HDAC6 and HDAC7 are involved in the expression of proinflammatory genes in macrophages stimulated by LPS.^100,101^ In fact, a specific subtype of HDAC7 (HDAC7-U) promotes the expression of TLR-induced inflammatory gene subsets, such as EDN1, IL-12p40, and IL-6.^101^ The inhibition of HDAC6 activity significantly limits LPS-induced macrophage activation and proinflammatory cytokines, regulated by the effects of HDAC6 on cell adhesion and microtubule acetylation.^100^ In addition, an integrated genomic approach shows that, in response to LPS, HDAC3-deficient macrophages are unable to activate almost half of the inflammatory gene-expression program.^102^

In addition to the histone modification associated with inflammatory genes directly, some mediators target TFs. SIRT1, a specific type of HDAC, suppresses macrophage activation through TFs such as p65, LXR, and IRF8.^103^ In LPS-activated macrophages, SIRT1 expression is downregulated, and the expression level of these TFs increased.

In addition to the direct combination of pro-inflammatory genes and the regulation of their transcription, regulators aimed at the suppression mechanisms are also required. As a methyltransferase, KMT6 (also known as EZH2) works on H3K27 as a key subunit of polycomb repressive complex 2.^104^ KMT6 directly targets suppressor of cytokine signaling 3 (SOCS3) to promote H3K27me3 and inhibits the expression of SOCS3, leading to uncontrolled TLR-induced NF-κB activation and increased proinflammatory gene expression, therefore playing important roles in regulating the inflammatory responses in macrophages. Moreover, the negative marker H3M27me3 generated by KMT6 persists after termination of the stimulus, and these inhibited genes no longer respond to the glucocorticoids IL-4 and M-CSF.^105^ In addition, the repression of M2 gene expression is also related to the downregulation of transcription factor MAF and MAF-binding enhancers, coordinated with the loss of LDTF binding and chromatin accessibility.^106^ Recently, it has been revealed that HDAC2 interacts with the c-Jun promoter and plays a key role in silencing c-Jun expression specifically, the activity of which is important for inhibiting the transcription of a series of inflammatory pathway genes, thereby enhancing proinflammatory gene expression indirectly.^107^ Moreover, HDAC9, interacting and colocating with many transcriptional repressors or corepressors, including NCOR13, during macrophage differentiation, represses these nuclear receptors by forming multiprotein complexes and thus induces a proinflammatory M1 phenotype.^108^

In addition to histone modification, chromatin remodeling occurs during M1 activation. Chromatin remodeling occurs through the ATP-dependent chromatin remodeling complex SWI/SNF, which is preferentially targeted to enhancers and serves as a histone acetylation sensor interacting with acetyltransferase P300 to modulate H3K27ac.^109^

DNA methylation affected by M1 activation is poorly investigated compared with histone modification and chromatin remodeling. A link between DNA methylation and LPS stimulation has been found for SOCS1, a negative regulator of cytokine signals.^110^ DNMT1-mediated hypermethylation of SOCS1 results in a loss of SOCS1 expression and enhances the release of LPS-induced proinflammatory cytokines such as TNF and IL-6 in macrophages.^110^ DNMT1 also induces the hypermethylation of the critical regulators Notch1, PU.1, and KIF4; participates in increasing the dimethylation (H3K9me2) and trimethylation (H3K9me3) of H3K9 in these genes; and skews their polarization towards M1 macrophages.^111^

Noncoding RNAs are also involved in M1 differentiation and the modulation of proinflammatory polarization. For example, a higher expression of proinflammatory miR-155 and a lower expression of miR-125b favor M1 macrophages.^112^ In contrast, mir-99a inhibits the phenotype and function of M1 macrophages by targeting TNF-α.^113^

### M2 alternative activation

Different from classical activation, the macrophages to be alternatively activated do not require a primed state, but appropriate inducers are needed, such as IL-4 and IL-13.

Transcriptional activation induced during M2 polarization is commonly associated with histone demethylase (Fig. 3). The increased expression of JMJD3, mediated by STAT6, contributes to transcriptional activation of M2 marker genes. Specifically, JMJD3 induces H3K27 demethylation at the transcription factor IRF4 locus, which is essential for M2 macrophage development and thus facilitates its expression.^114^ Accordingly, the JMJD3-IRF4 axis is necessary for M2 polarization in macrophages. Another study showed that αKG produced by glutamine lysis controls JMJD3-dependent regulation, suggesting the correlation of epigenetic and metabolic reprogramming for M2 macrophages.^115^ JMJD3 has been indicated to be a critical modifying enzyme in M2 polarization through JMJD3^−/−^ bone marrow chimeras, while the role in M1 activation is dispensable.^114^ The dual roles of JMJD3 in M1 and M2 macrophages are not inevitably conflicting, suggesting the need to remove the inhibitory H3K27me3 marker to respond to many environmental queues. Moreover, M2 polarization is also due to SMYD3 activity, another H3K4 methyltransferase.^95^ SMYD3 is preferentially expressed in M2 macrophages and can increase H3R4me3 at the M2-related promoter site.

With respect to deacetylases, the enzymes have both positive and negative effects. For instance, SIRT2 is an NAD^+^-dependent deacetylase that reduces NF-κB acetylation and increases M2-associated anti-inflammatory responses.^116^ In contrast, HDAC3 acts as an epigenomic brake on IL-4-induced alternative activation by deacetylating putative enhancers and thereby repressing IL-4-regulated genes characterized by M2 activation.^117^ Recently, IL-4-activated STAT6 was found to act as a transcriptional repressor in an HDAC3-dependent manner.^118^ The repressed inflammatory enhancers are associated with reduced LDTF and p300 binding followed by a reduction in enhancer RNA expression, H3K27 acetylation, and chromatin accessibility. Therefore, it can be inferred that HDAC3 plays an important role in M2 activation. Moreover, even without external stimulation, macrophages lacking HDAC3 exhibit an M2-like phenotype and are highly responsive to IL-4.^117^

DNA methylation and noncoding RNAs are also involved in macrophage alternative activation. DNMT3B modulates DNA methylation at the promoter of PPAR1, which is a key transcriptional factor that regulates macrophage polarization.^119^ Additionally, the expression of certain miRNAs, such as miR-142-5p and miR-511, promotes M2 polarization by negatively regulating genes involved in inflammatory signaling.^120,121^

To answer the question of whether the IL-4-activated macrophages are homogeneous, a recent study revealed that repeated stimulation with IL-4 induces strong phenotypic changes in macrophages through PPAR-γ, which has a significant ligand-insensitive, genome-bound fraction.^122^ After the first IL-4 exposure and subsequent STAT6 activation, PPAR-γ establishes a permissive chromatin environment through the recruitment of coactivator P300 and architectural protein RAD21, and this altered epigenome endows transcriptional memory by promoting the binding of STAT6 and RNA polymerase II. Therefore, the robust production of enhancer and mRNAs are induced upon IL-4 restimulation, and the expression of a hidden gene program, such as extracellular matrix gene network, reaches the threshold of activation only after the second stimulus.

### The role of epigenetic modification in macrophages during distinct disease states

Changes in macrophages can cause a broad spectrum of maladaptive immunity and inflammation that are causative factors of disease and thus represent key therapeutic targets.^4^ Moreover, the significant role of epigenetic pathways in macrophages in disease states indicates that epigenetic modifications of macrophages can be used for the diagnosis or therapies of indifferent diseases.

### Atherosclerosis

Atherosclerosis is a chronic inflammatory disorder as a result of a vascular injury caused by endothelial dysfunction. Macrophages are crucial immune cells in the progression of atherosclerosis and determining the clinical outcome through transmission inflammatory responses, foam cell formation and the final development of necrotic core.^123^ In the initiation of atherosclerosis, endothelial cells are activated by the aggregation of low density lipoprotein (LDL) and the modification of it (such as oxidation to oxLDL) in the arterial wall. Monocytes are attracted to adhere and migrate to the vessel wall and then differentiate into macrophages and may become lipid-loaded foam cells. Apoptotic foam cells can form the core of necrosis, and thus, the differentiation of monocytes into macrophages is a key step in the formation of atherosclerosis.

Abundant studies have focused on the epigenetic changes of macrophages involved in the pathogenesis of atherosclerosis, especially histone modification and drugs targeting atherosclerosis based on HDACs. It has been demonstrated that HDAC inhibitors have beneficial antiatherogenic effects as they partially inhibit M1 activation, reduce proinflammatory cytokine expression and blunt apoptosis; thus, they do not increase the formation of foam cells in primary macrophages. Valproate, a broad-spectrum HDAC inhibitor, has been shown to inhibit atherosclerosis in animal models.^124^ Nonspecific HDAC inhibitors, however, have contraindications that prevent their direct usage in the treatment of atherosclerosis. Therefore, specific HDAC enzymes deserve further study. HDAC3 has been detected to be a new potential target for atherosclerosis therapy, as deletion of HDAC3 can promote M2 activation while inhibiting M1 polarization.^123^ These two characteristics are considered to be the advantages of atherosclerosis therapy. Moreover, high expression of HDAC9 is consistent with an increased risk of atherosclerosis.^125^ HDAC9 upregulation in macrophages during atherosclerosis represses cholesterol efflux and M2 polarization, and in HDAC9-deficient mice, the phenotype of macrophages switches from the proinflammatory M1 to the anti-inflammatory M2 state via PPAR-γ.^108^

miRNAs are closely related to atherosclerosis in various aspects, including the regulation of macrophages during atherosclerosis. miR-155 plays a key role in macrophages, supporting and improving inflammation-induced atherosclerosis by directly repressing Bcl6.^126^ Moreover, it has been shown that miR-146a is involved in the pathogenesis of atherosclerosis, which negatively regulates macrophage maturation and inhibits inflammatory activation by reducing the expression of CD86 and CD80.^127^

### Obesity and type 2 diabetes

Obesity and type 2 diabetes (T2D) are rapidly growing diseases and are major risk factors for the development of cardiovascular diseases. Obesity generally tends to classically activate M1 adipose tissue macrophages and decreases alternatively activated M2 macrophages. The different activation states of M1 and M2 contribute to obesity-induced insulin resistance and inflammation.

In recent years, research has focused on the epigenetic mechanisms by which macrophages regulate their activation as crucial controllers of inflammation in T2D and therefore provide new insights into therapeutic interventions. Hyperlipidemia and hyperglycemia in diabetes patients could cause epigenetic changes and promote the formation of an inflammatory macrophage phenotype. In macrophages isolated from hyperlipidemia and a T2D mouse model of hindlimb ischemia, inflammatory genes are hypomethylated, and M2 genes are hypermethylated.^128^ In obesity patients, DNMT3B and DNMT1 expression are enhanced, leading to DNA methylation of the promoter of PPAR1, which may induce M2-associated gene suppression and contribute to an inflammatory macrophage phenotype.^129^ Therefore, the deletion of DNMT1 by using 5-aza-2′-deoxycytidine in pharmacology could promote alternative activation and inhibit macrophage inflammation. Furthermore, several lncRNAs are altered by T2D. One example is the lncRNA E330013P06, which is upregulated in macrophages isolated from T2D mice and the monocytes of T2D patients. The lncRNA E330013P06 may play an important role in macrophage dysfunction and related gene regulation because its overexpression in macrophages enhances the expression of inflammatory genes and increases inflammatory responses to specific signals.^130^

### Diabetic wound healing

Wound healing is a well-coordinated dynamic process involving diverse cells, including three main stages of coagulation and inflammation, tissue formation and remodeling. Macrophages are one of the critical participants in wound healing. Since the occurrence of injury, the functional changes of macrophages at the wound site persist. The major M1 subtype occurs mainly in the early inflammatory phase and is responsible only for the phagocytosis of bacteria, neutrophils, and tissue debris and the release of cytokines such as TNF-α and IL-6.^131^ As the repair/remodeling phase progresses, phenotypic transformation occurs in macrophages. During the late stage of repair, macrophages provide an M2 gene-expression profile predominantly, resulting in an increase in the number of M2 (healing-promoting) macrophages and the release of growth factors such as TGF-β, IL-10 and IGF.^131^ Delayed wound healing is a serious diabetes-related complication that often leads to nontraumatic limb amputations.

Given the role of epigenetic mechanisms in T2D, attention has recently been paid to epigenetic changes in diabetic wounds, and it has been revealed that a large number of epigenetic changes in diabetes lead to delayed wound healing. Alterations in histone methylation have been suggested to be a damaging factor for diabetic wound healing. The expression of IL-1β is increased in macrophages in diabetic wounds, and the abnormal expression of IL-1β impairs wound healing. After stimulation with LPS, the expression of IL-1β is increased in macrophages isolated from wounds of the T2D mouse model, with increased H3K4 methylation and decreased H3K27 methylation, suggesting that the expression of IL-1β is regulated by an epigenetic mechanism.^132^ It has recently been demonstrated that hyperglycemia leads to changes in the microRNA signature in wound healing, and they have been found to play a role in the dysregulated inflammation of diabetic wounds. For instance, miR-146a has been demonstrated to be downregulated in diabetic wounds and is unable to regulate its proinflammatory target gene expression, such as the NF-κB p65 subunit, leading to wound-healing impairment.^133^

### Sepsis

Sepsis is a worldwide disease with high morbidity and mortality rates, especially in intensive care units. Sepsis occurs when the body reacts to infectious and noninfectious stimuli that cause a nonresolving inflammatory response and cytokine release and then induce the injury of tissues and organs, leading to multiorgan failure, shock, and death.^134^ During sepsis, white blood cells and platelets migrate to the infection site, resulting in platelet aggregation, endothelial damage, and increased microvascular permeability. Blood flow also decreases, which may introduce ischemia-reperfusion injury. These physiological processes can induce systemic inflammatory response syndrome, leading to multiple organ dysfunction syndrome.

It is now well established that macrophages and other innate immune cells are activated profoundly during sepsis, playing a key role in the pathogenesis of this disease. In particular, dysregulated and profound activation macrophages can influence immune function and can directly affect the prognosis of sepsis.

Macrophage epigenetics plays an important role in the immune response associated with sepsis. For instance, the inhibition/modulation of HDACs can serve as a therapeutic approach for sepsis via modulating the epigenetic pathway. In particular, HDAC6 inhibitor has a substantial advantage in the treatment of sepsis.^135^ The selective inhibition can significantly reduce levels of proinflammatory mediators, inhibit macrophage apoptosis, promote bacterial clearance, increase immune cell phagocytosis, and improve survival in a lethal murine model of sepsis. The expression of microRNA-Let7A (let-7a) in patients with sepsis caused by gram-negative bacilli has been shown to be significantly downregulated.^136^ Let-7a has been confirmed to regulate the Toll-mediated inflammatory response in sepsis, thus providing a potential target for sepsis treatment. Recently, it has been demonstrated that lncRNA NEAT1 is upregulated in patients with sepsis and that overexpressed NEAT1 plays a key role in acute kidney injury induced by sepsis.^137^ Moreover, it has been found that the lncRNAs NEAT1/Let-7a and Let-7a/TLR4 have direct combinations. In the pathogenesis of sepsis, increased lncRNA NEAT1 binds to Let-7a competitively, and TLR4 is released from Let-7a, which is activated and stimulates downstream signals, leading to severe inflammatory responses.^138^

## Innate immune memory in macrophages

**Fig. 4:**
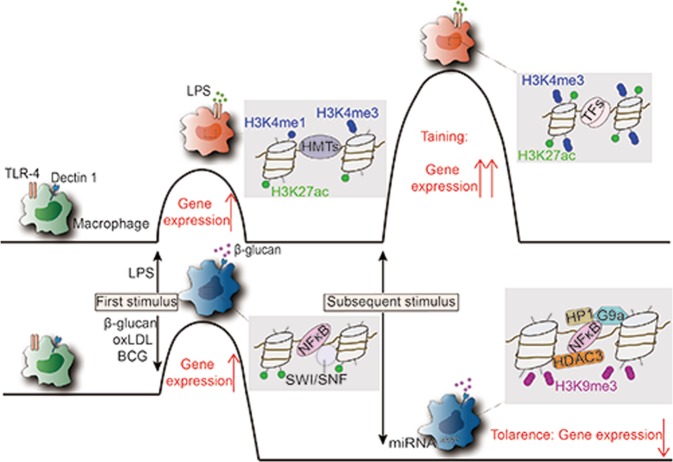
Trained immunity and tolerance are associated with epigenetic reprogramming. Macrophages can be trained or tolerized for specific inflammatory stimuli, depending on the type of trigger. In trained immunity, the initial hit induces long-lasting histone marks. In the case of the second hit, H3K27ac and H3K4me3 have already primed the genes, and the expression is enhanced. In contrast, in tolerance, the first stimulation leads to the activation of macrophages, but the removal of the stimulus results in the loss of the activating marks. In addition, suppressive nucleosome remodeling complexes and histone enzymes are involved in the process

Innate immune memory of macrophages is an important mechanism in response to environmental stimuli and affects subsequent immune responses and can be divided into tolerance and training. Recently, it has also been demonstrated that immune memory also occurs in tissue-resident macrophages in vivo, such as microglia.^139^ The molecular mechanisms responsible for memory-like activity in macrophages have not yet been elucidated. However, epigenetic regulations most likely induce these changes (Fig. 4).

### Tolerance

Acute inflammatory activation of macrophages induced by TLR and related receptors must be tightly regulated and transient to avoid tissue damage.^140^ The activation state is inherently unstable and is followed by a state of tolerance. Even in the continued presence of the agonist, the response to LPS is self-limiting. In murine macrophages, the TLR-induced response in vitro fell into two categories of LPS tolerance: the selective and transient silencing of proinflammatory genes and the priming of the second-class genes of M2 activation.^141^ Following the first exposure to LPS, anti-inflammatory genes in macrophages become modified to induce the second stimulation faster and stronger and thereby increase the efficiency of innate host defense. The consistent gene reprogramming that macrophages undergo is the result of complex regulatory mechanisms. For the molecular mechanism, LPS tolerance is mainly controlled by epigenetic regulation, including nucleosome remodeling, the reduced recruitment of transcription factors and chromatin remodeling complexes, and histone modification.^142^

Nucleosome remodeling complexes are essential in this process. Initial LPS stimulation of naïve macrophages is needed for the silencing of proinflammatory genes and the priming of anti-inflammatory genes in tolerant macrophages. LPS-induced gene products in naïve macrophages differentially modify chromatin at promoters of pro- and anti-inflammatory genes to silence the former and prime the latter through a second LPS stimulation. For example, BRG1 is recruited to the promoters of secondary response genes by the products of primary response genes, which explains why the induction of anti-inflammatory genes is qualitatively and quantitatively different in naïve and tolerant macrophages. Therefore, the SWI/SNF complexes, not for rapidly induced primary response genes, are required for the activation of primary response genes induced with delayed kinetics and secondary response. A Mi-2bβ complex, acting antagonistically to limit the induction of these gene classes, is selectively recruited along with the SWI/SNF complexes.^143^

The NF-κB-associated inhibitory mechanisms are essential in the tolerance process. NF-κB can recruit the NCOR-HDAC3-P50 repressive complex into targeted genes.^144^ Moreover, NF-κB can also recruit histone methyltransferase G9a to promoters to induce H3K9 methylation and induce binding of the heterochromatin protein 1.^145^ Together, these mechanisms lead to epigenetic silencing.

Noncoding RNAs, especially specific miRNAs, can regulate macrophage tolerization. For example, miRNA-146a has been shown to play a central role in TLR signaling tolerance following a primary stimulus with MyD88-dependent TLR pathways.^146^ Additionally, miRNA-221 and miRNA-222 are also found as regulators of the functional reprogramming of macrophages during LPS tolerization by regulating BRG1.^147^

### Trained immunity

The traditional view that only the adaptive immune system can build immunological memory has been challenged by a growing number of discoveries. In organisms lacking adaptive immunity, such as invertebrates, the innate immune system can mount long-term memory for resistance to reinfection.^148^ Studies that exposed macrophages to bacterial and fungal pathogens expand this observation, where the exposures enhance their subsequent response to the following stimulation with unrelated pathogens or PAMPs.^149^ Exposure of microglia to inflammatory stimuli can cause a long-lasting change or memory; when the microglia encounter subsequent inflammatory stimuli, they produce higher inflammatory responses.^150^ It has been demonstrated that trained immunity is accompanied by epigenetic reprogramming, especially histone modification.^151^ In response to stimulation, H3K4me1 levels and the binding of TFs increase at a subset of enhancers, named “latent enhancers”, and these enhancers do not return to a latent state when stimulation ceases, suggesting the establishment of an epigenetic memory that regulates cell responses to subsequent stimuli.^46^ For the promoter, after training in macrophages, an increased level of H3K4me3 can be observed at the promoter of genes associated with innate immunity, such as the adaptor molecule Myd88 and the proinflammatory cytokines TNF-α, IL-6, and IL-18, and it is a basis of robust transcriptional responses during trained immune responses.^149^

A low concentration of oxLDL treatment of monocytes can induce a long-lasting proatherogenic macrophage phenotype as “training”, enhancing H3K4me3 marks at promoter regions of these inflammatory genes, characterized by increased pro-inflammatory cytokine production.^152^ The molecular basis for this process has been found recently.

Trained immune genes through β-glucan and its receptor Dectin-1 are able to engage in chromosomal contacts with a subset of lncRNAs, and the upstream master lncRNA of the inflammatory chemokine locus can form chromosomal contacts with the ELR + CXCL chemokines (IL-8, CXCL1, CXCL2, and CXCL3) and *cis*-directs the WDR5/MLL1 complex across the CXCL chemokine promoters, facilitating their H3K4me3 epigenetic priming before their transcriptional activation.^153,154^

Innate immunity training plays an important role in disease prevention, among which research on Bacillus Calmette-Guérin (BCG) is a typical example. BCG can be used to train macrophages ex vivo and thereby provide cross-protection.^155^ Considering the relatively short lifespan, the ability of macrophages to transmit memory phenotypes to offspring and provide sustained protection remains unclear. In fact, the efficacy of generating long-term innate immune memory is essential to enhance organism immunity. Therefore, immune memory has also been studied for insights into HSCs, long-lived cells that are self-renewing and capable of producing multipotent and lineage-committed hematopoietic progenitors that give rise to all cells of the mammalian blood system, including macrophages.^156^ HSCs educated with BCG produce epigenetically modified macrophages, which are better than HSCs educated without BCG in preventing *M. tuberculosis* infection.^157^

Macrophages can be trained or tolerized for specific inflammatory stimuli, depending on the type of trigger. In trained immunity, the initial hit induces long-lasting histone marks. In the case of the second hit, H3K27ac and H3K4me3 have already primed the genes, and expression is enhanced. In contrast, in tolerance, the first stimulation leads to the activation of macrophages, but the removal of the stimulus results in the loss of the activating marks. In addition, suppressive nucleosome remodeling complexes and histone enzymes are involved in the process.

## Conclusion

Macrophages, heterogeneous cells whose surroundings regulate their phenotypes and functions, have central roles in danger detection, inflammation and host defense. Tissue-resident macrophages are specific to the environment and play a critical role in the maintenance of tissue homeostasis to maintain physiological functions. Under different transcriptional profiles and various stimulations, M1/M2 macrophages have almost opposite functions and different transcriptional profiles, but both of these cell types have unique abilities to destroy pathogens or repair inflammation-mediated damage; therefore, these cells are also necessary for restoring homeostasis. It is becoming increasingly clear that epigenetic modifiers have the ability to determine the fate of macrophages, whether in the health tissues or at the site of tissue injury. Therefore, the application of the epigenetic paradigm in macrophage studies is of great value. The dynamic regulation of epigenetic patterns in macrophages provides opportunities to alter disease-related epigenetic status. Hence, it can be assumed that understanding the epigenetic regulation of macrophages will substantially contribute to medicine in terms of diagnostic and therapeutic mechanisms and modalities.

Overall, work on the epigenetic regulation of macrophages has laid the foundation for the development of human medicine, and thus, this issue deserves more extensive and thorough study.
